# Network Toxicology and Molecular Docking Analysis of Targets and Potential Mechanisms of PEEK-Induced Bone Resorption

**DOI:** 10.3390/ijms27114709

**Published:** 2026-05-23

**Authors:** Yang Hu, Lei Zhang, Zhengbo Liu, Cailian Lu, Hong Li, Qiuying Yu, Sirui Lü, Lubin Liu, Junxing Liu

**Affiliations:** 1 School of Stomatology, Jiamusi University, Xiangyang District, Jiamusi 154000, China; 2 The Cell Biology Laboratory, School of Basic Medical Sciences, Southern Medical University, Shatai South Road, Baiyun District, Guangzhou 510515, China; 3 School of Basic Medicine, Jiamusi University, Xiangyang District, Jiamusi 154000, China; 4 School of Clinical Medicine, Jiamusi University, Xiangyang District, Jiamusi 154000, China

**Keywords:** polyetheretherketone, bone resorption, network toxicology, molecular docking, molecular dynamics simulation

## Abstract

Polyetheretherketone (PEEK), a high-performance thermoplastic, is utilized in bone tissue engineering due to its elastic modulus resembling that of human cortical bone. However, toxicological studies on PEEK remain limited. PEEK disrupts bone homeostasis by recruiting macrophages and inducing the aggregation of foreign body multinucleated giant cells, ultimately leading to bone resorption. The lack of effective therapeutic approaches underscores the importance of identifying novel treatments. This study systematically investigated the potential molecular mechanisms underlying PEEK-induced bone resorption using network toxicology, molecular docking techniques, and molecular dynamics simulations. We first conducted a network-based toxicological assessment based on the molecular structure of PEEK. By integrating and screening targets from multiple databases, we identified 139 potential targets associated with PEEK-induced bone resorption and constructed an interaction network diagram of these targets. Gene Ontology (GO)/KEGG enrichment analysis revealed that PEEK may induce bone resorption through pathways such as the PI3K-AKT signaling pathway and TNF signaling pathway. Further analysis using STRING and Cytoscape 3.9.0 software identified 53 core targets, including MAPK3, TNF, IL-6, AKT1, IL-1β, EGFR, and MMP9. We found that enriched highly correlated pathways encompassed core targets, supporting the scientific hypothesis that PEEK induces bone resorption. Furthermore, molecular docking and molecular dynamics simulation results confirmed that PEEK exhibits strong binding affinity with core targets, forming stable complexes. In summary, this study not only reveals the potential biological mechanisms underlying PEEK-induced bone resorption but also provides new evidence for future prevention and treatment of PEEK-induced bone imbalance.

## 1. Introduction

Network toxicology is an emerging discipline that focuses on the potentially adverse effects of chemical substances and the underlying molecular mechanisms by which they act. Built on the conceptual frameworks of network biology and network pharmacology, it leverages systematic network analysis and integrated database mining to identify candidate targets, infer toxic mechanisms and reveal critical regulatory relationships of exogenous compounds. By constructing predictive network models that describe and forecast the toxicological behaviour of chemicals, network toxicology aims to provide rational, mechanism-driven evidence for the safety assessment of compounds and biomaterials. Molecular docking is a computational chemistry technique that simulates molecular recognition by combining geometric (shape) and energetic (interaction) complementarity. Built on the physical principle of intermolecular interaction, it predicts the most probable binding modes by searching for ligand poses that minimize the binding free energy with a given receptor: geometric matching ensures spatial fit, whereas energetic matching sustains the stability of the bound state. Molecular dynamics (MD) simulation, in turn, applies physical force-field calculations to iteratively update the positions, velocities and forces of every atom in the system, thereby reproducing the time-evolved macroscopic behaviour and microscopic structure of biomolecules. MD makes it possible to evaluate whether a docked complex remains stable in an explicit aqueous environment and to characterize its dynamic equilibrium properties.

Patients with congenital tooth agenesis and postmenopausal individuals with orthopedic disorders frequently require surgical implants to replace native bone and restore physiological function [1]. Metallic and alloy materials are commonly chosen for such implants because they can mimic the mechanical performance of native bone [2] and are widely employed in orthopedic devices such as artificial joints and fracture-fixation hardware [3]. Despite these advantages, metallic implants present several well-documented limitations. First, biocompatibility issues can arise: even highly biocompatible materials such as titanium and titanium alloys may elicit local tissue and blood responses during long-term implantation [4], partly because corrosion and wear of the metallic components release metal ions into the surrounding tissues. Second, the wear resistance of metals is generally inferior to that of ceramic counterparts, and the abrasive particles generated by long-term articular friction can promote bone resorption and prosthesis loosening [5]. Third, hypersensitivity to metal ions—particularly to nickel and chromium—can produce clinical symptoms such as joint pain and swelling in susceptible patients [6]. Fourth, the high elastic modulus of metallic alloys gives rise to a “stress-shielding” effect, in which the implant absorbs a disproportionate share of the applied load and reduces the mechanical stimulation of the adjacent bone, thereby impairing periprosthetic bone formation [7]. Polymeric materials have therefore been proposed as implant substrates that can mitigate several of these issues [8].

Polyetheretherketone (PEEK), a high-performance thermoplastic polymer first approved by the U.S. Food and Drug Administration in the late 1980s [9], has attracted substantial attention because of its excellent mechanical performance, outstanding chemical resistance and high thermal stability [10]. Its elastic modulus is close to that of human cortical bone [11], which substantially mitigates the stiffness mismatch responsible for the stress-shielding effect observed with conventional metallic implants. PEEK is also radiolucent, producing minimal interference with medical imaging and thereby improving the postoperative diagnostic accuracy compared with metal alloys [12]. Its strong corrosion resistance ensures long-term in vivo stability, and its thermoplastic nature is fully compatible with three-dimensional (3D) printing, enabling patient-specific implants and establishing PEEK as one of the most widely investigated advanced polymers in 3D-printed orthopedic and dental applications [13].

Owing to these advantages, PEEK has progressively become a focus of bone-repair research and is regarded as a promising candidate for next-generation bone substitutes [14]. Nevertheless, several intrinsic limitations have constrained its broader clinical adoption. The principal concern is the inherent hydrophobicity of PEEK: its surface water-contact angle is typically 80–90° [15] and its biocompatibility at the cell–material interface is suboptimal [16]. This hydrophobic character impedes the direct adhesion of bone cells, particularly osteoblasts, and prevents the establishment of a productive osteogenic microenvironment around the implant. A fibrous capsule frequently develops at the implant–bone interface instead, predisposing to complications such as infection and pathological bone resorption and ultimately increasing the risk of surgical failure [17,18]. Although surface-modification strategies have markedly improved PEEK biocompatibility in recent years [19,20], the molecular mechanisms underlying PEEK-induced bone resorption remain poorly characterized. To systematically dissect the molecular targets and the putative biological pathways through which PEEK perturbs bone homeostasis, the present study integrates network toxicology, molecular docking and MD simulation. We anticipate that this multidisciplinary strategy will yield more comprehensive and in-depth scientific evidence for the safety assessment of PEEK and provide new perspectives on PEEK-related bone-resorptive disorders and possible interventional strategies.

## 2. Results

### 2.1. Chemical Information of PEEK

Basic chemical information of the PEEK structural repeat unit (monomer) is summarized in Table 1, providing the basis for the subsequent computational exploration of its potential interactions with human bone-resorption-related proteins. It should be emphasized that the descriptors listed correspond to the monomer of the PEEK polymer rather than to the bulk polymer chain.

### 2.2. Target Genes of PEEK

A total of 295 candidate target proteins associated with PEEK exposure were identified by combining the outputs of SwissTargetPrediction and COREMINE. These proteins represent the molecular nodes through which PEEK may exert biological effects after exposure and constitute the initial pool of candidates through which the toxicological consequences of PEEK—including responses related to bone resorption—could plausibly be mediated. Further analysis and experimental validation of these candidates will refine our understanding of the molecular mode of action of PEEK.

### 2.3. Identification of Target Genes Related to Bone Resorption and PEEK-Induced Bone Resorption

**Figure 1:**
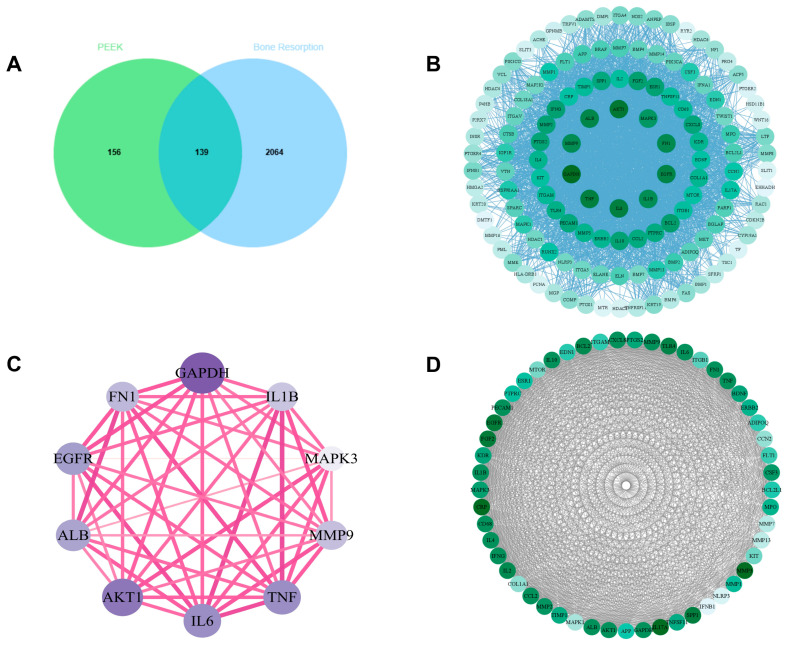
(**A**). Venn diagram of the targets of PEEK and bone resorption, (**B**). Visualization Results of Protein–Protein Interaction Network Analysis, (**C**). The top ten genes ranked by Degree value for PEEK-induced bone resorption were identified as core targets, (**D**). The region identified by MCODE analysis as having the strongest protein connectivity.

By querying the OMIM and GeneCards databases, 2203 targets strongly associated with bone resorption were identified. After integration and deduplication, intersection of the PEEK candidate list with the bone-resorption target repository yielded 139 overlapping targets (Figure 1A), which were taken as the putative effector set for PEEK-induced bone resorption. These 139 cross-over genes represent a focused molecular link between PEEK exposure and bone-resorptive pathology, and they were used as the input for all subsequent topological, functional and structural analyses. Downstream investigations were aimed at clarifying the specific contribution of each gene to the bone-resorption process and at evaluating their potential as therapeutic targets or biomarkers for assessing the risk of bone-homeostasis disruption associated with PEEK exposure.

### 2.4. Interaction Network of Potential Targets and Identification of Core Genes

A protein–protein interaction (PPI) network containing 135 nodes and 2666 edges, with an average node degree of 39.5, was constructed in STRING. Topological parameters of the network—degree, betweenness centrality and closeness centrality—were computed in Cytoscape, and a visually optimized network diagram (Figure 1B) was generated. Hierarchical clustering based on a composite centrality measure arranged the nodes into concentric rings, with the ten most central genes occupying the innermost ring. Ranking the nodes by degree value using the CytoHubba plug-in identified the top ten core targets associated with PEEK-induced bone resorption (Figure 1C). MCODE module analysis identified three densely connected sub-modules; the most densely connected sub-network, comprising 53 nodes and 1142 edges with a connectivity score of 43.923, is shown in Figure 1D and contains the targets with the most prominent topological influence on PEEK-induced bone resorption.

The PPI network constructed in Cytoscape graphically depicts the interactions among the candidate targets identified in this study. In the network diagram, node size and colour intensity scale with the degree value, so that hub targets are visualized as larger, more vividly coloured nodes; edge thickness and colour saturation likewise reflect the strength of the protein–protein associations. This visualization makes the topological prominence of and the functional links among the core targets immediately apparent, and inspection of the resulting connectivity pattern offers insight into the molecular interactions that may underpin PEEK-induced bone resorption.

### 2.5. Target Function Analysis and Pathway Enrichment Analysis

**Figure 2:**
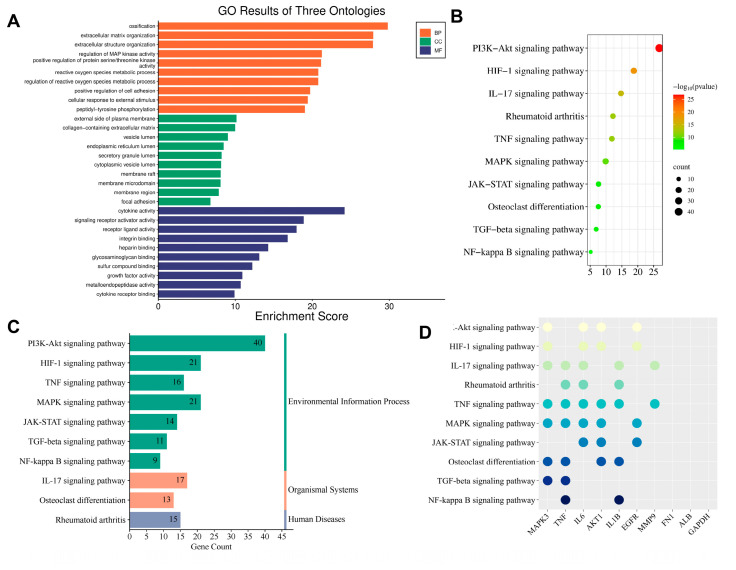
Analysis of GO and KEGG enrichment results. (**A**). Enriched biological processes (BP), molecular functions (MF), and cellular components (CC); (**B**). KEGG pathways related to bone resorption that are enriched with relevant genes; (**C**). Classification of the 10 enriched KEGG pathways; (**D**). Frequency of core genes in the enriched KEGG pathways related to bone resorption.

GO enrichment analysis of the 139 candidate targets, with the species restricted to humans, returned 4779 statistically significant GO terms—423 entries for molecular function (MF), 287 for cellular component (CC) and 4069 for biological process (BP). The ten most significant terms (smallest *p*-values) within each category were retained for the enrichment plot (Figure 2A). KEGG pathway analysis was then carried out on the same target set to identify the signaling cascades through which the candidate targets may operate. The unrestricted KEGG analysis returned a large number of statistically significant pathways, but most of them corresponded to general cellular processes or unrelated diseases that risked obscuring the bone-resorption-specific mechanisms of interest. To formulate a more focused mechanistic hypothesis, a targeted re-analysis was performed: the top ten enriched pathways (*p* < 0.05) directly relevant to osteoclast differentiation and bone metabolism were selected for visualization and presented as a categorical histogram and a statistical-significance bubble plot ordered by descending *p*-value (Figure 2B,C). The targeted analysis revealed a coherent functional module, indicating that the core targets do not converge on randomly distributed pathways but cluster on key regulators of bone resorption such as the PI3K–AKT signaling pathway, the TNF signaling pathway and osteoclast differentiation. This finding provides focused evidence that PEEK exposure may drive osteoclastogenesis through a defined set of signaling cascades, rather than through unrelated pathological processes, thereby disrupting bone homeostasis. Beyond pathway enrichment, the GO and KEGG results jointly link the candidate targets to biological processes including bone formation, reactive oxygen species (ROS) metabolism and cellular signaling—processes critical to the cellular response to external stimuli. Among the molecular functions, protein tyrosine kinase activity is conspicuously enriched and is well known to govern signaling pathways that regulate cell survival and proliferation. The cellular-component results highlight membrane rafts and cytoplasmic vesicular compartments, which are central to membrane trafficking and signal transduction. Within the bone-resorption-relevant subset of pathways, the PI3K–AKT signaling pathway emerges as the most prominent. A dot plot showing the ten most frequently recurring genes across these enriched pathways is presented in Figure 2D, in which seven core genes—MAPK3, TNF, IL-6, AKT1, IL-1β, EGFR and MMP9—are highlighted; each dot indicates the participation of a given gene in a particular enriched pathway, and the overall pattern reflects the strength of association between each gene and the enriched pathway set. On the basis of this convergent topological and functional evidence, MAPK3, TNF, IL-6, AKT1, IL-1β, EGFR and MMP9 were prioritized as the seven hub genes for the downstream structural analyses.

### 2.6. Molecular Docking Results

**Figure 3:**
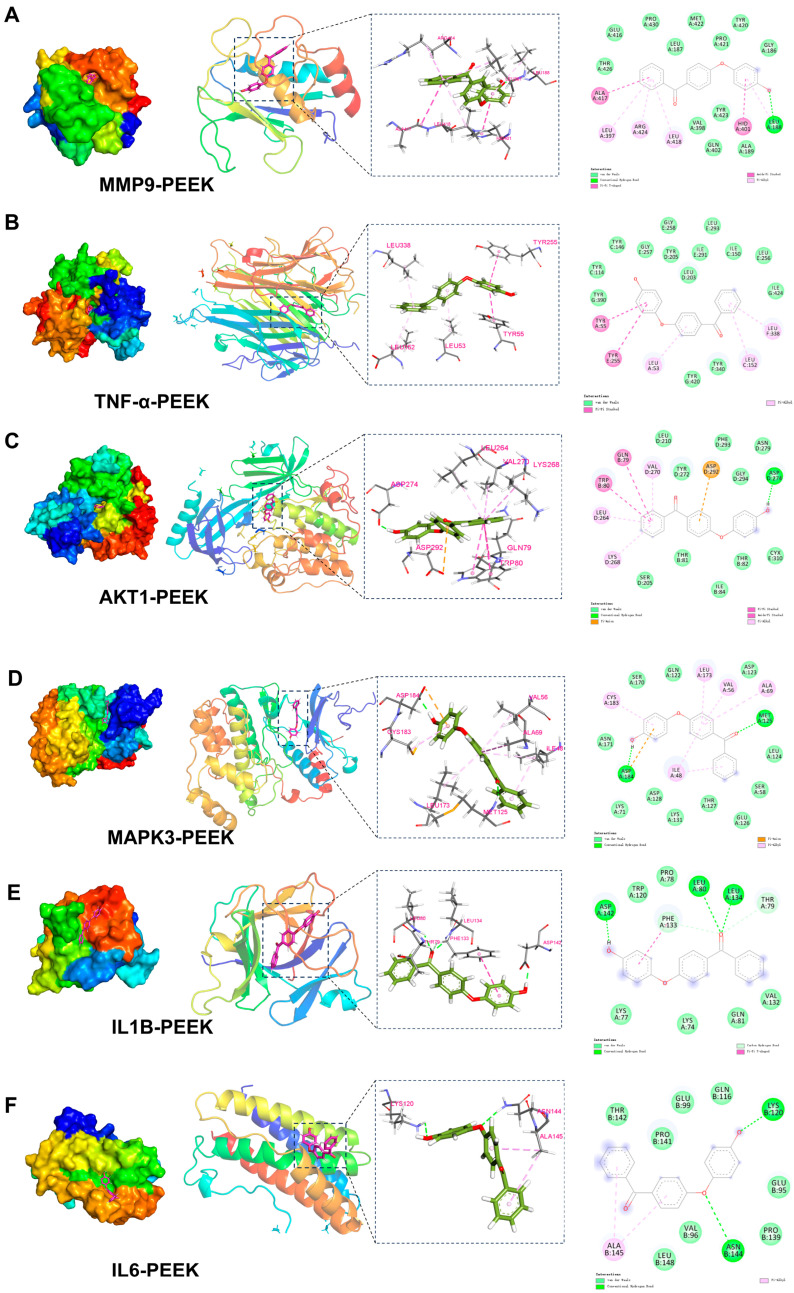

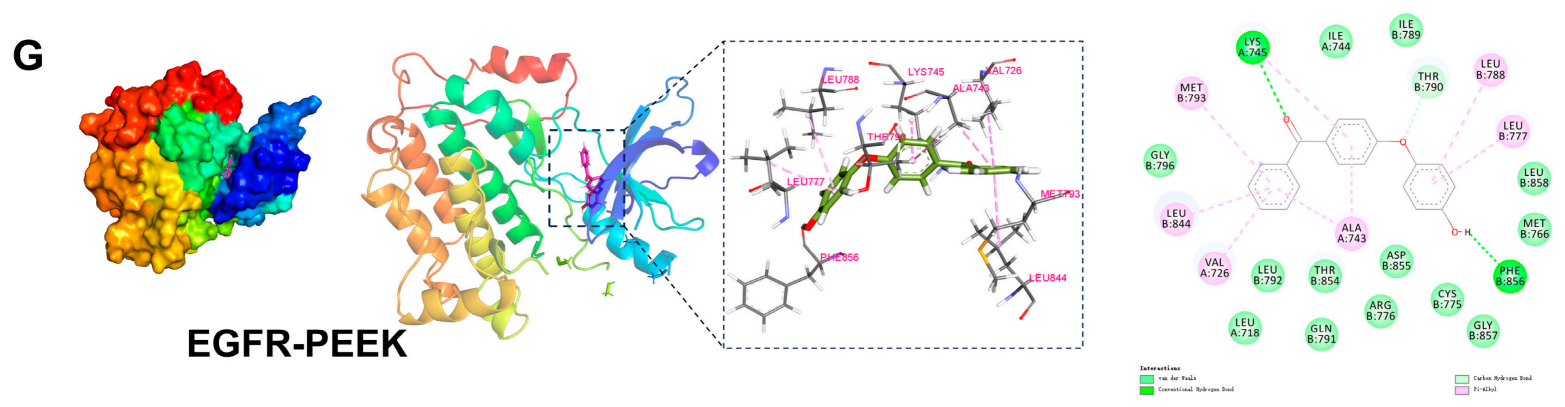
Visualization of the molecular docking of PEEK to the core target protein. (**A**) Molecular docking of MMP9 with PEEK. (**B**) Molecular docking of TND-α with PEEK. (**C**) Molecular docking of AKT1 with PEEK. (**D**) Molecular docking of MAPK3 with PEEK. (**E**) Molecular docking of IL-1β with PEEK. (**F**) Molecular docking of IL-6 with PEEK. (**G**) Molecular docking of EGFR with PEEK. Optimal binding conformation with lowest affinity (kcal/mol) is shown. The protein backbone is shown in cartoon or surface form, and the PEEK ligand is highlighted in a stick model. The dotted line indicates intermolecular interactions, including van der Waals, Conventional Hydrogen Bond, Pi-Anion, Pi-Pi Stacked, Amide-Pi Stacked, Pi-Alkyl and Carbon Hydrogen Bond.

Molecular docking was performed between PEEK and the seven hub proteins MAPK3 (PDB ID: 6GES), TNF-α (7KP9), IL-6 (1ALU), AKT1 (4EJN), IL-1β (5R88), EGFR (8PO4) and MMP9 (2OW1) (Figure 3, Table 2). All seven complexes returned binding free energies more negative than −6 kcal mol^−1^, indicating consistent and energetically favorable docking between PEEK and each core target. The lowest binding energy was observed for the PEEK–MMP9 complex (−10.6 kcal mol^−1^), in which π–π stacking interactions, van der Waals contacts and conventional hydrogen bonds jointly stabilized the bound state, whereas PEEK–IL-6 displayed the weakest, but still favorable, binding (−6.4 kcal mol^−1^).

### 2.7. Molecular Dynamics Simulation

The structural conformations and dynamic equilibrium of the PEEK–protein complexes were further evaluated by 100 ns all-atom MD simulations of the seven hub-target complexes. Five complementary descriptors—RMSD, Rg, RMSF, ligand–protein distance and buried solvent-accessible surface area (buried SASA)—were monitored throughout each trajectory, and an additional reduced trajectory containing one snapshot per nanosecond (101 frames in total, denoted traj_0–100) was extracted from each simulation for visual confirmation of the conformational behaviour of both the protein and the bound PEEK molecule.

**Figure 4:**
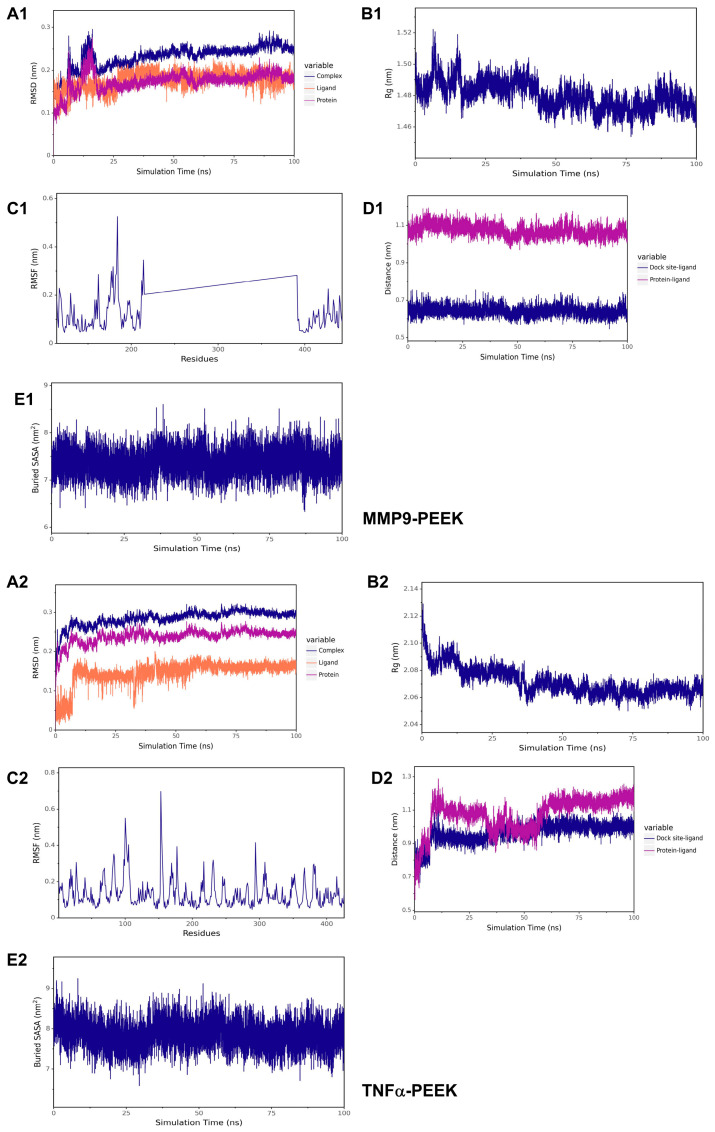

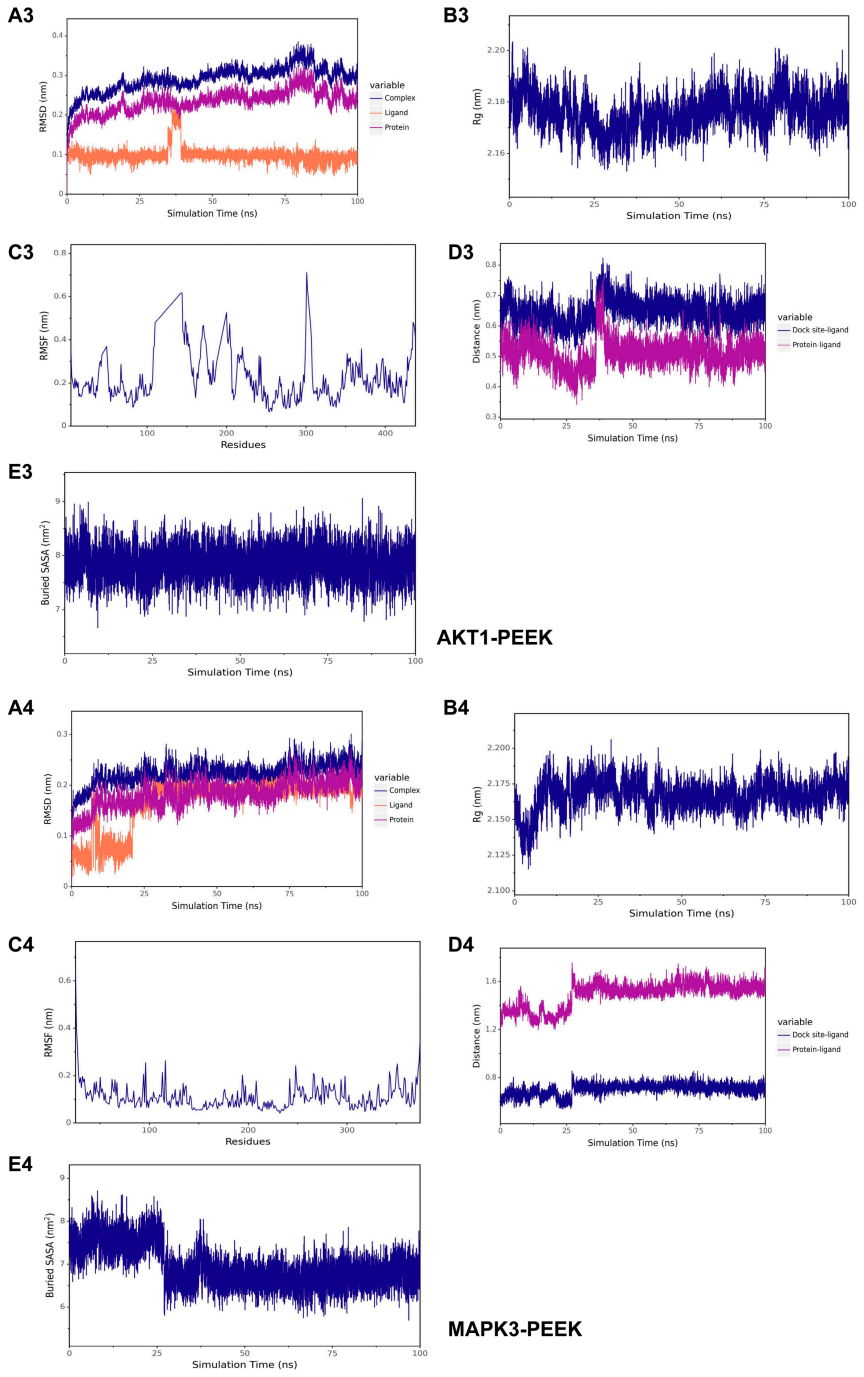

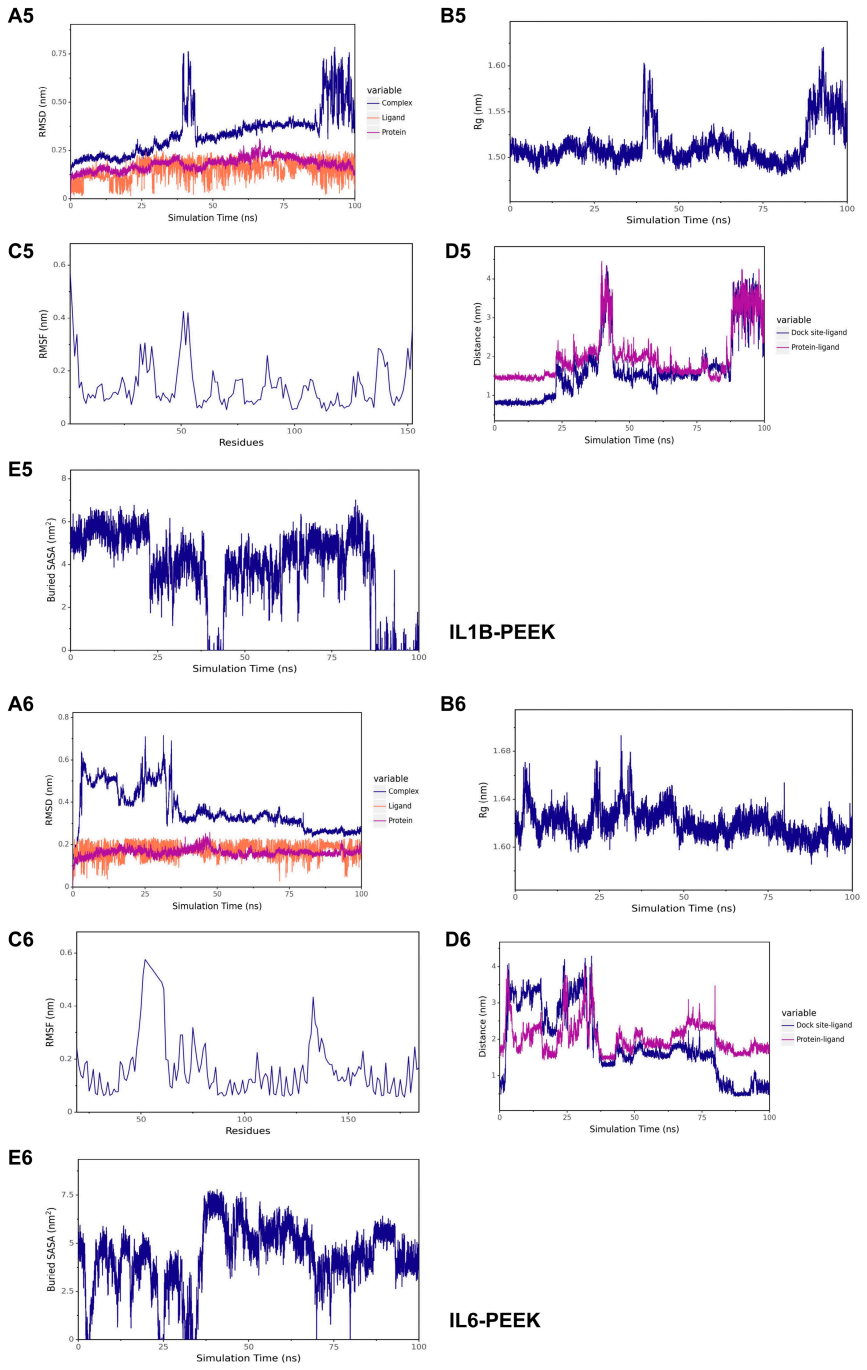

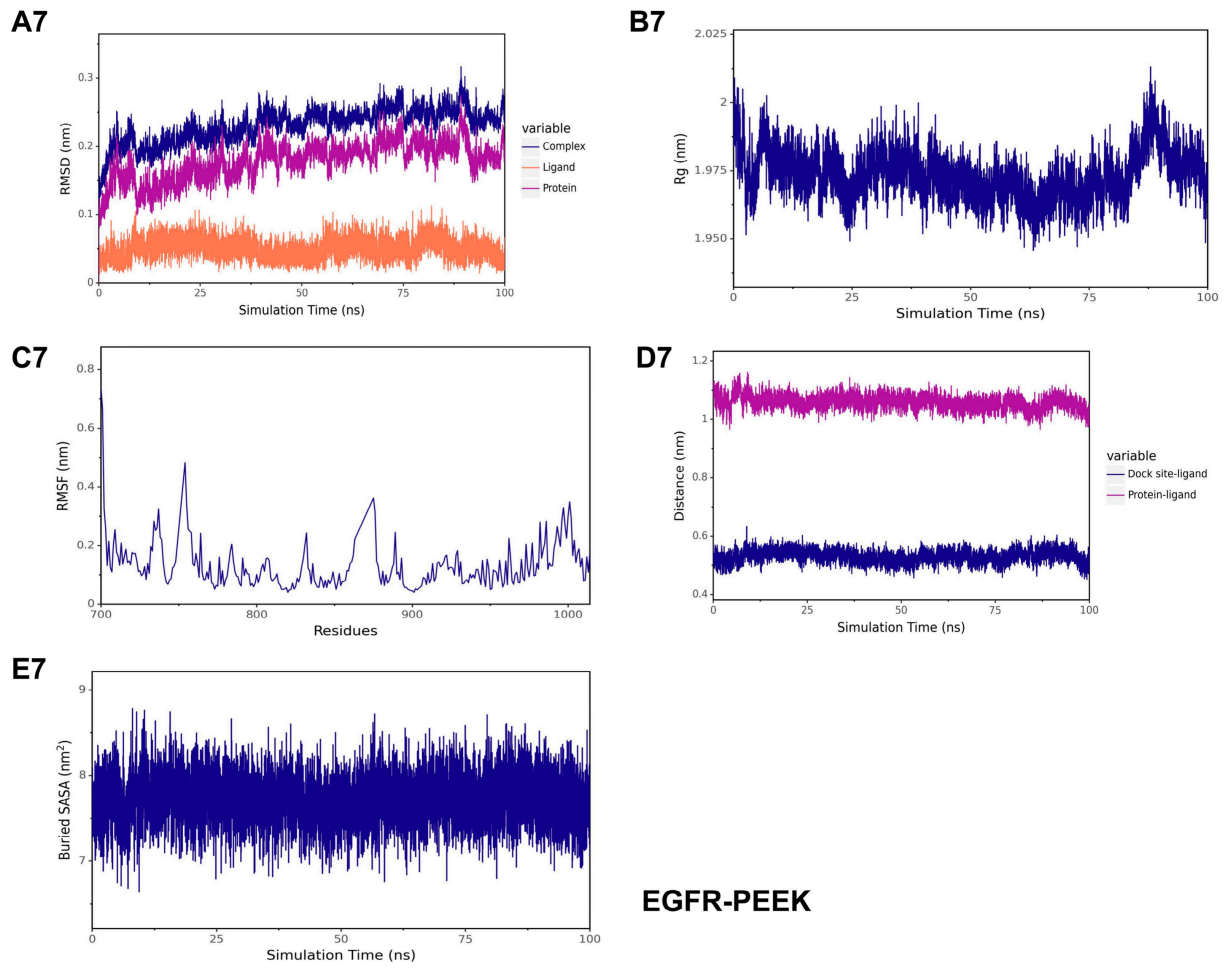
Molecular dynamics simulation results for each PEEK–core target complex. (**A**) Root Mean Square Deviation, RMSD. (**B**) Radius of Gyration, Rg (**C**) Root mean square fluctuation, RMSF. (**D**) Distance. (**E**) Buried Solvent Accessible Surface Area, Buried SASA. (**A1**–**E1**) Molecular Dynamics Simulation of MMP9 with PEEK. (**A2**–**E2**) Molecular Dynamics Simulation of TNF-α with PEEK. (**A3**–**E3**) Molecular Dynamics Simulation of AKT1 with PEEK. (**A4**–**E4**) Molecular Dynamics Simulation of MAPK3 with PEEK. (**A5**–**E5**) Molecular Dynamics Simulation of IL-1β with PEEK. (**A6**–**E6**) Molecular Dynamics Simulation of IL-6 with PEEK. (**A7**–**E7**) Molecular Dynamics Simulation of EGFR with PEEK.

The root-mean-square deviation (RMSD) is the most widely used measure of structural similarity between two configurations, and the magnitude of its plateau during MD reflects the degree of conformational equilibration relative to the starting structure. As shown in Figure 4A, both the protein-only RMSD and the complex RMSD progressively converged for all seven systems within the 100 ns time scale, indicating that the simulated complexes reached a structurally stable regime. For the IL-1β–PEEK and IL-6–PEEK complexes, transient several-Ångström excursions were observed in the all-atom RMSD trace during the early phase of the trajectory; visualization of the corresponding traj_0–100 snapshots confirmed that these excursions originated from the motion of solvent-exposed flexible loops and intrinsically disordered terminal segments of the cytokine fold—most notably the long N-terminal extension of IL-6, which is unresolved in the parent crystal structure 1ALU and is widely predicted to be intrinsically disordered—whereas the secondary-structure cores adjacent to the PEEK-binding interface remained well superimposed throughout the simulation. To formally substantiate this interpretation, a binding-pocket-restricted RMSD was additionally computed using only the Cα atoms of residues within 5 Å of PEEK at t = 0; this restricted RMSD remained below 0.20 nm throughout the entire 100 ns for both complexes, confirming that the binding interface itself is conformationally stable.

The radius of gyration (Rg) measures the root-mean-square distance of all atoms from the molecular centre of mass and provides a global descriptor of the structural compactness of a protein–ligand complex: a smaller Rg corresponds to a more compact fold, whereas an inflated Rg suggests a looser, partially unfolded geometry. As shown in Figure 4B, the Rg of every complex stabilized within the 100 ns trajectory, indicating that the overall packing of each complex remained compact and that no large-scale unfolding events occurred during the simulations.

Per-residue root-mean-square fluctuation (RMSF) values quantify the amplitude of atomic motion around the time-averaged position over the trajectory and thereby identify the most rigid and most flexible regions of each protein. Discontinuities at residue indices that correspond to homology-rebuilt loops were rendered as breaks rather than as interpolated lines so that the RMSF profiles unambiguously reflect physically meaningful residue-level dynamics. As anticipated, elevated RMSF values were concentrated on solvent-exposed loops and chain termini, whereas the secondary-structure cores—and particularly the residues in direct contact with PEEK—displayed only modest fluctuations (Figure 4C), consistent with stable engagement of the binding interface.

To monitor the spatial relationship between PEEK and the protein, the time-resolved distances between the centres of mass of the ligand and the binding-site residues, and between the ligand and the protein as a whole, were tracked. As shown in Figure 4D, both distances stabilized in every complex within the 100 ns time scale. For the TNF-α–PEEK, IL-1β–PEEK and IL-6–PEEK systems, modest step-like changes in the distance trace were observed during the first 30–50 ns; visualization of the corresponding traj_0–100 snapshots demonstrated that PEEK did not dissociate into the bulk solvent in any of these systems but rather adjusted its position within the same surface-exposed region, settling into a chemically equivalent neighbouring sub-pocket dominated by aromatic and hydrophobic residues. This local reorientation is consistent with the small molecular weight of the PEEK monomer (328.31 g mol^−1^) and with the shallow surface character of typical cytokine binding sites, and per-frame contact analysis confirmed that PEEK maintained close contact (<4 Å) with multiple key interaction residues identified in the docking pose throughout the second half of the simulation. The plateaued distances and the persistent contact pattern therefore indicate that PEEK reaches a dynamically stable bound state in each system rather than undergoing dissociation.

The buried solvent-accessible surface area (buried SASA) reports the surface area of the complex that is shielded from solvent through ligand binding, with larger values reflecting a larger protein–ligand contact area and stronger intermolecular association. As shown in Figure 4E, the buried SASA of each complex stabilized over the 100 ns trajectory, indicating that PEEK and its target protein established a sustained and well-defined contact surface and that the strength of the interaction did not deteriorate during the course of the simulation.

Taken together, the trajectory descriptors and the snapshot-based visualizations indicate that all seven PEEK–core-target complexes attained progressive structural equilibration: the protein and complex RMSDs converged to plateau values, the Rg of each complex remained compact, the ligand–protein distances and the buried SASA stabilized, and PEEK was consistently retained within either the original docking pocket or a chemically equivalent neighbouring sub-pocket throughout the second half of the simulations. These results provide robust dynamic evidence for the formation of stable PEEK–core target complexes and corroborate the docking-derived binding patterns presented in Section 2.6.

## 3. Discussion

PEEK has emerged as one of the most actively investigated polymers in the field of bone repair and reconstruction, where it is increasingly used to replace or fix damaged bone elements [21]. Its broad indications—from edentulous dental implant abutments to filling materials for bone defects—mean that a substantial population of patients with bone-repair needs is exposed to this material [22]. Despite its compelling materials-science profile, the intrinsic hydrophobicity of PEEK and its prolonged in vivo residence time have raised concerns about its potential to disturb bone homeostasis. In particular, growing attention is being paid to the link between long-term PEEK exposure and increased peri-implant bone resorption [23]. Experimental evidence indicates that prolonged contact between PEEK-based bone-repair materials and the in vivo bone microenvironment can promote local bone resorption at the repair site, and that this risk may be amplified in vulnerable populations such as patients with osteoporosis [23]. While the inherent hydrophobicity of PEEK hinders the direct adhesion of osteoblasts [24] and impairs biological osseointegration, the same hydrophobic character paradoxically drives strong non-specific protein adsorption [25]. According to well-established principles of biomaterial surface science, when hydrophobic materials are implanted in vivo or generate wear debris, their surfaces immediately interact with the surrounding biological fluids [26] and rapidly adsorb host proteins to form a “protein corona”. The adsorbed proteins frequently undergo conformational changes that expose cryptic epitopes recognized by the host immune system as foreign-body antigens [27], thereby acting as potent stimuli for osteoclast differentiation. The molecular cascade linking these events to peri-implant bone loss, however, remains incompletely characterized. Building on recent computational toxicology work that addressed analogous problems—such as the effect of DOTP exposure on periodontitis [28]—the present study integrated network toxicology, molecular docking and MD simulation to examine the molecular pathophysiology of PEEK-induced bone resorption. Network toxicology, which combines bioinformatics, systems biology and chemoinformatics, is well suited to capturing how an exogenous compound perturbs cellular function and disrupts biological networks toward disease-relevant phenotypes. Molecular docking estimates the atomic-level binding patterns and affinities between PEEK and bone-resorption-related target proteins, while MD simulation provides a theoretical foundation for assessing whether these interactions are dynamically stable in an aqueous environment. By integrating these complementary approaches, our analysis seeks to contribute new computational evidence to the broader discussion of PEEK safety and its role in the maintenance of bone homeostasis. Within this framework, intermolecular contact distance, contact persistence, atomic displacement and the temporal similarity of bound conformations are pivotal observables: contact analysis yields the geometry and chemical specificity of the interaction, whereas MD-derived parameters report on its stability over time. On the basis of the integrated network and structural results, seven hub genes—MMP9, TNF-α, AKT1, EGFR, MAPK3, IL-1β and IL-6—were highlighted as the principal molecular nodes through which PEEK is predicted to disturb bone homeostasis.

MMP9 is a matrix metalloproteinase capable of degrading multiple extracellular matrix components, notably collagen and gelatin. Its expression and activity are tightly regulated by the RANKL signaling axis, and MMP9 is a critical effector in osteoclast-mediated bone resorption [29]. A wealth of skeletal-biology studies have shown that MMP9 is markedly overexpressed in pathological conditions such as osteoporosis and inflammatory bone deterioration [30], and that pharmacological inhibition of MMP9 substantially reduces collagen breakdown and bone-matrix degradation [31]. Several independent in vivo studies have corroborated this mechanism. Mechanistically, MMP9 is essential for the initiation and maintenance of bone resorption: it degrades bone-matrix components such as type I collagen and facilitates the migration of osteoclast precursors toward mineralized bone surfaces [32,33]. TNF-α is a powerful inducer of bone resorption both in vitro and in vivo. It promotes osteoclast differentiation and activity directly and indirectly enhances bone resorption by suppressing osteoblast functions [34], including collagen synthesis [35], alkaline phosphatase (ALP) activity [36] and bone-specific glycoprotein (BGP, osteocalcin) production [37]. As a master coordinator of immune responses, TNF-α plays roles in both host defense and pathological inflammatory injury, and skeletal-system studies have repeatedly documented a strong correlation between TNF-α and inflammatory bone loss [38]. TNF-α antagonists are clinically effective in suppressing inflammation and slowing bone destruction [39], whereas TNF-α levels are markedly elevated in disorders such as rheumatoid arthritis [40]. Numerous preclinical investigations have produced reproducible mechanistic findings: TNF-α directly promotes the survival and differentiation of osteoclast precursors [41], synergizes with RANKL to amplify osteoclastogenesis [42], and indirectly drives osteoblastic expression of pro-osteoclastic cytokines [43], thereby disrupting the balance of bone remodeling and triggering pathological bone resorption.

AKT1, a downstream effector of the cAMP–PKA system, governs cellular fate decisions including survival, apoptosis and oncogenic transformation [44]. Studies of bone and cartilage tissue have established a strong relationship between AKT1 activity and inflammatory responses [45]: the AKT1 pathway is downregulated during inflammation, and elevation of AKT1 phosphorylation substantially attenuates the cytotoxic consequences of inflammatory stress [46]. AKT1 is also indispensable for the maintenance of bone-remodeling homeostasis, modulating osteoblast apoptosis, osteoclast maturation and the bone-resorption program [47].

EGFR, a receptor tyrosine kinase activated by ligands of the epidermal growth factor family, plays an essential role in the control of cell survival, proliferation and differentiation, and its aberrant activation is closely associated with tumor initiation and progression [48]. Recent skeletal-system studies have linked EGFR signaling to bone loss in malignant bone metastases. In the bone-metastatic microenvironment of solid tumors—such as breast adenocarcinoma and prostate carcinoma—tumor-derived EGF-family ligands can activate EGFR on osteoclasts via paracrine signaling [49], thereby indirectly enhancing their bone-resorptive capacity. The direct contribution of EGFR to physiological bone remodeling, however, remains a matter of debate. Notably, in selected models, preclinical studies have shown that EGFR inhibitors such as gefitinib partially attenuate bone loss [50], although this protective effect is most likely attributable to tumor suppression rather than to a direct action on bone cells. EGFR signaling can also act indirectly by upregulating key pro-resorptive mediators such as RANKL and PTHrP in tumor cells [51], thereby destabilizing bone remodeling and contributing to pathological bone resorption.

MAPK3 is a core member of the MAPK signaling pathway family and a major regulator of cell survival, differentiation, proliferation and stress responses to extracellular cues. Skeletal-biology studies have firmly established that MAPK3/ERK1 signaling is intimately linked to bone metabolic homeostasis [52]. Phosphorylation-dependent activation of MAPK3 is persistently upregulated during RANKL-induced osteoclastogenesis, and inhibition of its upstream MEK kinase markedly suppresses osteoclast differentiation and reduces bone resorption [53]. The convergent findings of multiple genetic and pharmacological investigations corroborate this mechanism. MAPK3 also drives osteoclastogenesis by activating downstream RANKL-responsive transcription factors such as c-Fos [54] and may indirectly influence bone remodeling by modulating osteoblast function—a key axis in pathological bone loss.

IL-6, a multifunctional cytokine secreted by immune and stromal cells, is essential for orchestrating inflammatory and immune responses, including pathological inflammatory damage and host defense. Skeletal-system studies have demonstrated a strong link between IL-6 and pathological bone loss, including postmenopausal and inflammatory osteoporosis [55], and IL-6 levels are markedly elevated in diseases such as rheumatoid arthritis and conditions of estrogen insufficiency [56,57]. Blockade of the IL-6 signaling axis—for example, with the IL-6 receptor antagonist tocilizumab—effectively slows bone destruction in patients [58]. Mechanistically, IL-6 mainly enhances osteoclast survival and differentiation indirectly, by promoting soluble RANKL release and upregulating RANKL expression in osteoblasts and stromal cells [59]; it also modulates osteoblast activity through the JAK–STAT signaling pathway [60], a regulatory contribution central to its capacity to disturb bone-remodeling homeostasis and promote pathological bone resorption.

IL-1β is a potent pro-inflammatory cytokine produced primarily by macrophages and a number of other cell types [61], and is one of the principal drivers of host inflammatory and immune responses. Its overproduction is directly implicated in pathological injury and several autoimmune disorders. Skeletal-system studies have shown that IL-1β is one of the key mediators of inflammatory bone deterioration: in rheumatoid arthritis, for example, synovial IL-1β levels are markedly elevated, and blockade of its signaling with IL-1 receptor antagonists such as anakinra substantially reduces bone erosion in both patients and experimental animals [62]. Multiple independent studies have replicated this conclusion. Mechanistically, IL-1β directly activates NF-κB and MAPK signaling on osteoclast precursors, thereby synergistically enhancing RANKL-driven osteoclastogenesis and survival [63]; it also indirectly stimulates the expression of pro-resorptive factors such as prostaglandin E2 and RANKL in osteoblasts and synovial fibroblasts. This dual action contributes both to the disruption of bone homeostasis and to the marked exacerbation of pathological bone resorption.

Collectively, these findings indicate that PEEK-induced bone resorption may involve a coordinated network of signaling cascades centered on MMP9, TNF-α, AKT1, EGFR, MAPK3, IL-1β and IL-6 (Figure 2). The well-documented roles of these molecules in cellular survival, immune signaling, osteoclast differentiation and matrix degradation provide multiple complementary perspectives on the biological mechanisms by which PEEK may disturb bone homeostasis. They also highlight a series of candidate target pathways for the development of more effective therapeutic strategies aimed at preventing peri-implant PEEK-induced bone resorption, and the relationships among these genes and pathways offer attractive directions for future research and intervention.

Our enrichment results, which converge on inflammation-related signaling, are consistent with prior reports underscoring the role of pro-inflammatory mediators in shaping the peri-implant inflammatory milieu [64]. Within this framework, MMP9, TNF-α, AKT1, MAPK3, IL-1β and IL-6 may collectively orchestrate the inflammatory component of bone resorption and could serve as informative biomarkers or therapeutic targets, with the influence of the TNF signaling pathway on the inflammatory environment and on cell survival being particularly relevant. Molecular docking is, in this context, a computational technique that simulates how complex compounds engage their target proteins; tighter contacts and more extensive interactions are reflected in more favorable (more negative) docking energies. The docking results (Figure 3, Table 2) indicate that the binding affinity between PEEK and the identified core targets, including MMP9 and TNF-α, is dominated by hydrophobic and π–π stacking interactions: the repeating aromatic rings of the PEEK structure can insert deeply into hydrophobic pockets such as the S1′ subsite of MMP9 and the dimer-interface pocket of TNF-α [65,66], engaging in stable π–π contacts with aromatic residues such as tyrosines. Hydrogen bonds donated by the ketone group provide additional anchoring, but the overall stability of the bound state is largely governed by the polycyclic aromatic backbone. The binding free energies of PEEK with these core targets ranged from −6.4 to −10.6 kcal mol^−1^, with PEEK–MMP9 displaying the most favorable energy (−10.6 kcal mol^−1^, Table 2). This is consistent with previous evidence that MMP9 overexpression contributes to the disruption of bone-homeostasis processes [67].

Drawing on these integrated computational results, we propose the following possible coordinated signaling scheme for PEEK-induced bone resorption. Micro- and nanoscale wear particles or oligomers released from PEEK material during long-term in vivo service can directly engage transmembrane receptors such as EGFR on host cells—including macrophages and osteoclast precursors—triggering receptor autophosphorylation and initiating downstream signal transduction [50]. Activated EGFR then recruits and activates parallel kinase networks: it phosphorylates AKT1 via the PI3K–AKT pathway to support the survival of osteoclast precursors, and concurrently phosphorylates MAPK3 (ERK1) via the MAPK pathway to promote nuclear translocation of osteoclast-differentiation transcription factors [68,69]. AKT1 and MAPK3 in turn activate transcription factors such as NF-κB and AP-1 [70], driving large-scale transcription and secretion of the potent pro-osteoclastic and pro-inflammatory cytokines TNF-α, IL-1β and IL-6 [71]. TNF-α further sustains a paracrine loop through the TNF signaling pathway [72], recruiting additional monocytes and synergizing with RANKL to lower the threshold for osteoclast differentiation [73,74], while IL-1β and IL-6 amplify the inflammatory milieu and further promote osteoclastogenesis [75]. Under this self-reinforcing signaling and inflammatory environment, monocytes and macrophages fuse to form mature osteoclasts that express and secrete large amounts of MMP9 [76], which degrade the type I collagen network of the bone matrix in the acidic micromilieu of the resorption lacuna [77] and ultimately drive the substantial peri-implant bone resorption and prosthesis loosening observed clinically around PEEK implants.

The present study has several limitations that should be acknowledged, even though it offers new insights into the relationship between PEEK and bone resorption. Currently available docking algorithms are optimized for small-molecule ligands and cannot routinely accommodate true high-molecular-weight polymers [78,79]. PEEK itself is highly biocompatible and resistant to chemical or enzymatic degradation, but under clinical conditions mechanical wear of PEEK implants generates micro- and nano-sized debris [80]. To approximate the chemical features of these wear-derived fragments, we used the PEEK monomer as a simplified surrogate, with its ether and ketone functional groups providing a plausible representation of the surface chemistry of nanoscale PEEK debris that would interact with host proteins. Although simulation of longer oligomeric fragments would more faithfully reproduce the polymer chain, the monomer model is sufficient for an initial estimation of binding affinity. While this fragment-based approach captures the principal local non-covalent interactions between PEEK functional groups and target proteins—such as π–π stacking and hydrophobic contacts—it cannot fully reproduce the macroscopic dynamics of the bulk-polymer/protein interface. Future studies that employ longer oligomer models or interface-resolved MD methods will be required to validate and extend the present findings. Comprehensive longitudinal clinical investigations that monitor disease progression and assess the susceptibility of different patient subgroups to PEEK exposure will further help to establish causal relationships and to mitigate adverse outcomes.

A second methodological caveat concerns the structural completeness of the parent crystal structures used for the MD simulations. Several of the deposited PDB entries contain unresolved residues annotated under the REMARK 465 field, most notably AKT1 (PDB ID 4EJN), in which two longer segments (residues 110–143 and 188–200) were not modeled in the original X-ray experiment. To minimize the risk that these gaps would introduce artificial chain breaks or unphysical residue-level dynamics during MD, missing fragments of meaningful length were rebuilt by homology modeling against the corresponding full-length human reference sequence prior to topology generation, whereas short surface loops located far from the PEEK-binding interface (≥15 Å Cα–Cα separation) were retained as in the deposited structures. The locally restricted nature of the missing segments and their physical separation from the modeled binding pockets ensure that the PEEK-binding analyses presented above are not biased by the incompleteness of the experimental structures. We further verified the conformational behaviour of each complex by visualizing every nanosecond snapshot of the 100 ns trajectory; in every system, the protein backbone in the binding region remained well superimposed across snapshots, and PEEK either occupied the original docking pocket or settled into an immediately adjacent, chemically equivalent surface sub-pocket. Nevertheless, longer time-scale simulations using fully reconstructed full-length protein models or AlphaFold-predicted starting structures will help to further consolidate the present dynamic conclusions.

Although the present network-toxicology and structural analyses successfully delineated a 139-target predictive network and prioritized a panel of core biomarkers (most notably MAPK3, IL-6, TNF-α and MMP9), an additional limitation of this work is the absence of immediate in vivo or in vitro experimental confirmation. To rigorously establish whether these targets are differentially regulated under bona fide PEEK exposure, future studies should adopt standardized biological models.

We therefore propose a comprehensive in vitro validation pipeline based on macrophage and osteoblast co-culture systems. Specifically, murine macrophage cell lines (such as RAW264.7) or primary bone-marrow-derived macrophages (BMMs) should be exposed to clinically relevant sizes and concentrations of PEEK wear particles to mimic the peri-implant microenvironment. The following readouts can validate the present predictions: (i) cytokine secretion—the upregulated transcription and extracellular release of early pro-inflammatory targets, principally TNF-α and IL-6, should be quantified by reverse transcription quantitative PCR (RT-qPCR) and enzyme-linked immunosorbent assay (ELISA), respectively; (ii) kinase cascade activation—activation of the predicted signaling hubs, in particular phosphorylation of MAPK3 (ERK1/2) and components of the PI3K–AKT pathway, should be evaluated by Western blotting; and (iii) pathological endpoint—the ultimate functional consequence, namely osteoclast differentiation and activity, can be assessed by tartrate-resistant acid phosphatase (TRAP) staining and bone-resorption pit assays carried out in the presence of PEEK-conditioned macrophage-conditioned media. Coupling such empirical assays with the present computational predictions will translate the bioinformatic signal into clinically actionable therapeutic targets for the prevention of PEEK-induced osteolysis.

In summary, this study clarifies key channels through which PEEK may compromise the dynamic balance between osteoblasts and osteoclasts and outlines the gene-expression patterns and inflammatory responses that may emerge from these interactions, providing a basis for the rational design of tailored therapeutic strategies to mitigate the risk of PEEK-induced disruption of bone homeostasis.

## 4. Method

### 4.1. Acquisition of PEEK Chemical Composition Information

The chemical composition of PEEK was retrieved from the PubChem (https://pubchem.ncbi.nlm.nih.gov/, accessed on 22 March 2025) and Google Scholar (https://scholar.google.com/, accessed on 22 March 2025).

### 4.2. Collecting PEEK Targets

The PEEK structure was retrieved from PubChem using the search term “polyether ether ketone”. The corresponding SDF file was downloaded and uploaded to the NovoPro database (https://www.novopro.cn/, accessed on 24 March 2025) to generate the canonical SMILES string. Based on this representation, putative PEEK targets were collected from the relevant literature and from the SwissTargetPrediction (http://www.swisstargetprediction.ch/, accessed on 26 March 2025) and COREMINE Medical (https://coremine.com/medical/, accessed on 26 March 2025) databases, with the species restricted to “Homo sapiens”. Targets retrieved from the two databases were merged and deduplicated, and gene symbols were normalized against the UniProt Knowledgebase (https://www.uniprot.org/, accessed on 29 March 2025). The resulting standardized list constituted the PEEK target library used in subsequent analyses.

### 4.3. Identification of Bone Resorption-Related Targets

Bone-resorption-related targets were collected by combining a comprehensive literature search with database queries against GeneCards (https://www.genecards.org/, accessed on 1 April 2025) and OMIM (https://www.omim.org/, accessed on 5 April 2025) using the keyword “bone resorption”. To minimize the risk of omission, all genes returned from each database were exported and integrated, and gene symbols were standardized against the UniProt Knowledgebase to build a bone-resorption target repository. A Venn diagram was then used to identify the intersection between the PEEK target library and the bone-resorption target repository; this intersection was defined as the candidate target set for PEEK-induced bone resorption.

### 4.4. Construction of Protein Interaction Networks

A protein–protein interaction (PPI) network among the candidate targets was constructed using the STRING platform (https://string-db.org/cgi/input.pl, accessed on 10 April 2025). The minimum required interaction score was set to “medium confidence (>0.4)” and the species was restricted to “Homo sapiens”, in order to retain biologically meaningful interactions among the input target genes. The resulting interaction file was imported into Cytoscape 3.9.0, a network biology visualization and analysis platform, where the PPI network was constructed and visualized and the topological parameters of every node were calculated.

### 4.5. Core Target Screening

Core targets for PEEK-induced bone resorption were defined as nodes that simultaneously satisfied three criteria: (i) Degree greater than twice the network median, (ii) Betweenness Centrality above the network median and (iii) Closeness Centrality above the network median. A composite centrality score was then computed by aggregating these three metrics so as to capture the overall topological importance of each node, and genes were ranked in descending order of their composite scores. The MCODE plug-in was used to identify the most densely connected modules within the PPI network.

### 4.6. Gene Function and Pathway Enrichment Analysis of Target Proteins

To dissect the biological functions of the candidate targets in PEEK-induced bone resorption, Gene Ontology (GO) and Kyoto Encyclopedia of Genes and Genomes (KEGG) pathway enrichment analyses were performed. GO analysis was carried out across three categories—molecular function (MF), cellular component (CC) and biological process (BP)—to identify the principal biological functions of the input gene set. KEGG analysis was used to identify signaling pathways significantly enriched among the candidate targets, with particular attention paid to pathways relevant to bone metabolism and osteoclast differentiation. The enrichment results were visualized using bar charts and bubble plots so that category, gene count and statistical significance could be appreciated simultaneously. This integrated functional annotation was intended to clarify the principal biological processes and pathways through which PEEK may disrupt bone health, thereby refining mechanistic insight into PEEK-induced toxicity.

### 4.7. Molecular Docking

Molecular docking was performed to estimate the binding modes and affinities of PEEK toward the prioritized core targets. The three-dimensional structure of the PEEK monomer was retrieved as an SDF file from PubChem and energy-minimized in Chem3D 22.0.0. Crystal structures of the target proteins were obtained from the Protein Data Bank (PDB; https://www.rcsb.org/, accessed on 2 May 2025) and pre-processed in PyMOL 3.1 by removing crystallographic water molecules and co-crystallized ligands. The cleaned protein structures were then prepared in AutoDock Tools 1.5.7 by assigning Gasteiger charges, adding polar hydrogen atoms and merging non-polar hydrogens. After the docking grid box had been defined around the experimentally annotated active or ligand-binding site of each target and appropriate evolutionary algorithm parameters had been selected, docking was performed using AutoDock Vina 1.5.7 [78] from the command line. The docking poses were ranked by their binding free energies, and the lowest-energy pose for each complex was retained for visualization in PyMOL (https://pymol.org/, accessed on 10 May 2025) and used as the starting structure for subsequent MD simulation.

### 4.8. Molecular Dynamics Simulation

PEEK and the protein structure with the lowest molecular docking free energy for each core target were used as the starting configuration for the MD simulation. Prior to topology generation, every protein crystal structure was inspected for unresolved residues annotated under the REMARK 465 field of the corresponding PDB file (4EJN for AKT1, 2OW1 for MMP9, 5R88 for IL-1β, 1ALU for IL-6 and 7KP9 for TNF-α). Short surface fragments (≤4 consecutive residues) located far from the modeled binding interface (≥15 Å Cα–Cα separation) were retained as in the deposited structures, whereas longer missing segments—most notably residues 110–143 and 188–200 of AKT1, residues 1–18 (the disordered N-terminal extension) and 52–60 of IL-6, and residues 102–111 of two TNF-α chains—were rebuilt by homology modeling against the corresponding full-length human reference sequence (UniProt entries P31749 for AKT1, P14780 for MMP9, P01584 for IL-1β, P05231 for IL-6 and P01375 for TNF-α) using the loop-modeling protocol implemented in MODELLER. The rebuilt models were energy-minimized in vacuum and visually checked for stereochemical quality before being passed to GROMACS 2025.2. This pre-processing step ensured that the simulated systems were free of artificial chain breaks and that subsequent residue-level analyses—particularly RMSF—could be unambiguously assigned to physically meaningful positions.

Molecular dynamics simulations were performed in GROMACS 2022 [81]. Small-molecule ligand parameters were generated under the GAFF force field [82], whereas the proteins were modeled with the AMBER ff14SB force field [83] in conjunction with the TIP3P water model [84]. The protein and ligand topologies were combined to assemble each complex system, which was then immersed in a cubic box of TIP3P water with a minimum distance of 1.0 nm between the solute and the box edges, neutralized with sodium and chloride ions to a physiological ionic strength of 0.15 M, and energy-minimized using the steepest-descent algorithm. Periodic boundary conditions were applied throughout the simulations, with constant temperature (V-rescale, 298 K) and pressure (Berendsen, 1 bar) coupling. All bonds involving hydrogen atoms were constrained using the LINCS algorithm [85], allowing an integration time step of 2 fs. Long-range electrostatic interactions were evaluated using the Particle-mesh Ewald (PME) method with a 1.2 nm real-space cutoff [86], the non-bonded neighbour list was updated every ten steps and short-range non-bonded interactions were truncated at 1.0 nm. Following 100 ps of NVT and 100 ps of NPT equilibration, each complex was subjected to a 100 ns production run at 298 K, with conformations saved every 10 ps for analysis. To enable visualization-based assessment of the conformational behaviour of each complex, an additional reduced trajectory containing one snapshot per nanosecond (101 frames in total) was extracted in parallel. Trajectory analyses—including the root-mean-square deviation (RMSD) of the protein backbone and the complex, the radius of gyration (Rg), the per-residue root-mean-square fluctuation (RMSF) of Cα atoms, the ligand–binding-site and ligand–protein-centre distances and the buried solvent-accessible surface area (buried SASA)—were performed with the GROMACS tools 2025.2, and the corresponding structural visualization was carried out in PyMOL and VMD 1.9.4 [87].

## 5. Conclusions

The present study delineates a plausible molecular link between PEEK exposure and bone resorption, providing important new information to inform PEEK surface modification and bone-immunology strategies. Our analyses offer a comprehensive perspective on how PEEK may influence the progression of bone resorption and underscore the need to reassess the long-term risks of PEEK use, while at the same time identifying potential therapeutic avenues for mitigating PEEK-induced bone loss. We expect that the prioritized core targets and pathways will stimulate further mechanistic investigation of PEEK-related bone-resorptive pathology and accelerate the development of effective preventive and therapeutic interventions to limit the adverse effects of PEEK-associated bone-homeostasis disruption.

## Figures and Tables

**Table 1:** Chemical formula, SMILES structure, and molecular weight of PEEK.

Chemical Information of PEEK	
Chemical Formula	C_19_H_14_F_2_O_3_
SMILES Structure	*OC1=CC=C(OC2=CC=C(C=C2)C(=O)C2=CC=C(*)C=C2)C=C1
MW (g/mol)	328.31

**Table 2:** Binding energy for target with PEEK.

Complex_Name	Affinity (kcal/mol)
MMP9-PEEK	−10.6
TNF-PEEK	−10
AKT1-PEEK	−9.5
EGFR-PEEK	−9.5
MAPK3-PEEK	−8
IL-1β-PEEK	−6.8
IL-6-PEEK	−6.4

## Data Availability

The original contributions presented in this study are included in the article. Further inquiries can be directed to the corresponding authors.

## Abbreviations

PEEK	Polyetheretherketone
